# Synergistic Activation of Innate and Adaptive Immune Mechanisms in the Treatment of Gonadotropin-Sensitive Tumors

**DOI:** 10.1371/journal.pone.0061288

**Published:** 2013-04-08

**Authors:** Anjali Bose, Ilpo Huhtaniemi, Om Singh, Rahul Pal

**Affiliations:** 1 Immunoendocrinology Lab, National Institute of Immunology, New Delhi, India; 2 Department of Physiology, University of Turku, Turku, Finland; 3 Department of Reproductive and Developmental Biology, Imperial College, London, United Kingdom; University of Strathclyde, United Kingdom

## Abstract

Human chorionic gonadotropin (hCG) prolongs the secretion of progesterone from the corpus luteum, providing a critical stimulus for the sustenance of pregnancy. hCG (or individual subunits) is also secreted by a variety of trophoblastic and non-trophoblastic cancers and has been associated with poor prognosis. Early clinical studies have indicated merit in anti-hCG vaccination as potential immunotherapy, but anti-tumor efficacy is believed to be compromised by sub-optimal immunogenecity. In the present study, enhanced tumorigenesis was observed when SP2/O cells were subcutaneously injected in either male or female BALB/c x FVB/J^βhCG/-^ F1 transgenic mice, establishing the growth-promoting effects of the gonadotropin for implanted tumors *in vivo*. The utility of *Mycobacterium indicus pranii* (*MIP*) was evaluated, as an innate anti-tumor immunomodulator as well as adjuvant in mice. *MIP* elicited the secretion of the inflammatory cytokines IFNγ, IL-6, IL-12p40, KC and TNFα from murine antigen presenting cells. When *MIP* was incorporated into an anti-hCG vaccine formulation previously employed in humans (a βhCG-TT conjugate adsorbed on alum), elevated T cell recall proliferative and cytokine responses to hCG, βhCG and TT were observed. *MIP* increased vaccine immunogenicity in mice of diverse genetic background (including in traditionally low-responder murine strains), leading to enhanced titres of bioneutralizing anti-hCG antibodies which exhibited cytotoxicity towards tumor cells. Individual administration of *MIP* and βhCG-TT to BALB/c mice subcutaneously implanted with SP2/O cells resulted in anti-tumor effects; significantly, immunization with βhCG-TT supplemented with *MIP* invoked synergistic benefits in terms of tumor volume, incidence and survival. The development of novel vaccine formulations stimulating both adaptive and innate anti-tumor immunity to induce collaborative beneficial effects may fill a niche in the adjunct treatment of hCG-sensitive tumors that are resistant to conventional therapy.

## Introduction

Human chorionic gonadotropin (hCG), a heterodimeric glycoprotein hormone, is considered crucial for the establishment of pregnancy. It is initially secreted by the pre-implantation embryo and later by the placental trophoblast. In its best-studied role, hCG “rescues” the corpus luteum from degeneration, leading to the sustained release of progesterone from the ovary, which in turn prepares the uterus for implantation. Besides its pregnancy-sustaining effects, hCG is increasingly recognized as a cancer marker of broad utility [1]. It is ectopically expressed by a wide variety of cancers of trophoblastic and non-trophoblastic origin and its presence is frequently associated with poor prognosis [2], [3], possibly as a consequence of chemo-resistance [4], [5]. Most cancers secrete the β-subunit, although secretion of the α-subunit and of the holo hormone can also occur. The growth-promoting properties of hCG are possibly attributable to the fact that the subunits contain a “cysteine knot” structural motif [6], a feature also found in nerve growth factor, platelet derived growth factor and transforming growth factor β. As the roles of hCG (and its subunits) in angiogenesis and cellular proliferation are being elucidated [7], [8], it is appears a promising target for cancer immunotherapy.

In humans, immunological tolerance to hCG can be overcome by conjugation to carrier molecules (such as tetanus toxoid or diphtheria toxoid) as has been established in clinical trials carried out by our lab [9]–[11]. Earlier efficacy studies with vaccines targeting the βhCG subunit (employed either as a contraceptive measure in women [9] or as immunotherapy in patients of colorectal cancer [12]) provided proof of safety and efficacy. It was surmised, however, that more immunogenic formulations could result in enhanced benefit. Though considered safe, alum (employed in most human vaccines as adjuvant) is frequently incapable of enhancing immune responses to desirable levels; in particular, immune mechanisms that require Th1 responses, or the elicitation of CD8+ T cells (for example, responses to virally-infected or transformed cells), are frequently compromised due to the Th2 skew that alum mediates [13]. There is continuous effort to develop newer formulations that bolster anti-tumor immunity without inducing toxicity, and bacterial components are worthy of consideration in this regard. In particular, *Mycobacterium indicus pranii* (*MIP*) has been shown to decrease tumor volume, with associated improvement in lung function in patients of lung cancer [14]. *MIP* has also been found to provide some clinical benefit in patients of bladder cancer [15].

In this study, the effects of *MIP* on antigen presenting cells (studied as components of splenic cells, as purified macrophages and as bone marrow derived dendritic cells) were first ascertained. Using heterozygous male and female βhCG transgenic mice, this study unequivocally established the growth-promoting effects of βhCG/hCG on implanted, histocompatible tumor cells. It further describes the benefits of *MIP* as a supplemental additive to an anti-hCG vaccine formulation previously tested in humans, both as an adjuvant for the enhanced generation of anti-hCG immune responses, as well as an elicitor of independent innate anti-tumor immunity. Towards this end, murine strains of several different haplotypes were immunized with βhCG-TT, with or without supplemented *MIP*. The influence of the inclusion of *MIP* on subsequent T cell recall responses towards individual vaccine components was determined. Serum anti-hCG antibody responses were characterized for titre, isotype and bio-neutralization capacity. Further, the ability of elicited antibodies to bind to, as well as mediate cytotoxicity of, tumor cells was assessed. Finally, the effects of *MIP* supplementation of the anti-hCG vaccine formulation were assessed in mice implanted with syngeneic tumors. Results revealed that *MIP* can have a variety of immuno-modulatory effects, and co-administration along with active anti-hCG immunization can result in substantial reductions in tumor incidence and volume, as well as enhancement in life-span.

## Materials and Methods

### Ethics Statement

This study was carried out in strict accordance with the protocol approved by the Institutional Animal Ethics Committee (IAEC) of the National Institute of Immunology, New Delhi (IAEC Number: 231/10). Blood samples were withdrawn under ketamine and xylezine anaesthesia and all efforts were made to minimize suffering.

### Animals

The generation and characterization of βhCG transgenic mice has been previously described [16], [17]. Wild-type BALB/c females were mated with heterozygous FVB/J βhCG transgenic males (FVB/J^βhCG/-^). F1 mice were bred at the animal facility of National Institute of Immunology, New Delhi, as were other inbred murine strains (BALB/c:H-2d; CBA:H-2k; C57BL/6:H-2b; FVB:H-2q; C3H-HeJ:H-2k).

### Assessment of the tumor-promoting activity of βhCG/hCG *in vivo*

PCR was performed on genomic DNA isolated from BALB/c x FVB/J^βhCG/-^ F1 animals using a forward primer corresponding to the ubiquitin promoter (5′-CGCGCCCTCGTCGTGTC-3′) and a reverse primer corresponding to βhCG (5′-AAGCGGGGGTCATCACAGCTC-3′). The presence of an 830 bp product indicated the presence of the transgene. To confirm productive transgenesis in F1 mice, serum βhCG was quantified by radioimmunoassay. Briefly, hCG standards (1.25, 2.5, 5, 10, 20 and 40 ng/ml), or sera at varying dilutions, were incubated at 4°C for 18 hrs with a murine anti-hCG monoclonal antibody in the presence of ^125^I-hCG (approximately 10,000 cpm; specific activity: 40–60 µCi/ µg) and 4% v/v normal horse serum. Antigen-antibody complexes were precipitated by the addition of 12.5% w/v PEG followed by centrifugation at 1500 g at 4°C for 20 min. Radioactivity in the pellet (comprising the antibody-bound fraction) was assessed. The concentration of βhCG in sera was estimated with reference to the standard curve.

Transgenic and non-transgenic BALB/c x FVB/J^βhCG/-^ F1 mice of both sexes were subcutaneously implanted with 10^4^ SP2/O cells. Tumor volumes were measured every alternate day using the formula 4/3πr^3^ where r  = (l+w)/4, using the largest axis of the tumor (l, length; w, width).

### Mycobacterium indicus pranii (*MIP*)

#### Culture

*MIP* was grown in Middlebrook 7H9 media (BD DIFCO) supplemented with 10% v/v albumin-dextrose complex enrichment (BD DIFCO), 0.02% v/v glycerol, and 0.05% v/v Tween 80. Mycobacteria were harvested by centrifugation at 840 g for 15 min. Bacterial concentrations were estimated spectrophotometrically. Bacteria were killed by autoclaving at 121°C at a pressure of 15 lb/in^2^ for 20 min.

#### Influence on the maturation of bone marrow-derived dendritic cells

Bone marrow cells procured from the femur and tibia of naive C57BL/6 and BALB/c mice were induced to differentiate in presence of GMCSF (50 ng/10^6^ cells) for six days. Replenishment with GMCSF-supplemented medium occurred on the third day. Resultant BMDCs were stimulated *in vitro* with different concentrations of *MIP* lysate. Levels of TNFα, KC, IL-12p70 and IL-6 in culture supernatants were determined by ELISA (eBiosciences).

#### Influence on cytokine secretion by macrophages

Macrophages were purified from spleen cells (derived from naive C57BL/6 mice) by magnetic sorting (Miltenyi Biotec) using biotinylated anti-CD11b antibodies (BD Biosciences) to 96% purity. Following incubation of macrophages and macrophage-deficient spleen cells with 1 µg/ml *MIP* lysate for 48 hr, the levels of TNFα and IL-6 in the culture supernatants were quantified by ELISA (eBiosciences).

### βhCG-TT conjugation and immunization

βhCG was conjugated with TT, essentially using a strategy previously described [18], and the βhCG-TT conjugate adsorbed on Alhydrogel (Superfos, Denmark). In initial experiments, βhCG-TT was employed at a range of doses. The effects of supplementation with live, heat-killed or autoclaved *MIP* (derived from two different sources, at doses ranging from 10^5^ to 10^7^ equivalent bacteria per injection) on immunogenicity were also assessed. The subcutaneous and intramuscular routes of immunization (with βhCG-TT and *MIP* either co-administered or administered in different limbs) were additionally compared. Several of these experiments were carried out in two murine strains to ensure broader validity. Based on the data generated, mice of different strains (BALB/c:H-2d; CBA:H-2k; C57BL/6:H-2b; FVB:H-2q; C3H-HeJ:H-2k) (n = 16) received three intramuscular injections of either the conjugate (2 µg βhCG equivalent) or the conjugate supplemented with *MIP* (10^7^ bacteria equivalent) at fortnightly intervals.

### T cell recall responses

4×10^5^ spleen cells from immunized and non-immunized mice were individually stimulated with hCG, βhCG, TT or *MIP*. ^3^H-thymidine (1 µCi/well) was added to cultures at 72 hr and cellular thymidine uptake was assessed on a liquid beta scintillation counter 16 hr later. In parallel experiments, IFN-γ, IL-5, TNFα and IL-6 were estimated by ELISA (eBiosciences) in supernatants collected at 48 hr.

### Characterization of antibody responses

#### Anti-hCG antibody titres

Antibody titres were estimated by a direct-binding radioimmunoassay [18]. Briefly, diluted sera obtained from immunized mice were incubated with a fixed quantity of ^125^I-hCG (approximately 10,000 cpm; specific activity: 40–60 µCi/ µg) and 4% v/v horse serum at 4°C for 48 hr. The antigen-antibody complex was precipitated by addition of 12.5% w/v polyethylene glycol (Sigma; Mw 8000) and centrifugation at 1500 g at 4°C for 20 min. Radioactivity in the pellet was determined in a gamma counter. Titres were expressed as hCG binding capacity per ml of undiluted serum.

#### Bioneutralization capacity of elicited anti-hCG antibodies

The capacity of the antibodies to inhibit the binding of ^125^I-hCG to rat testicular receptors was assessed [11]. Briefly, rat testicular homogenate was incubated with varying dilutions of antisera and ^125^I-hCG (approximately 30,000 cpm; specific activity: 40–60 µCi/ µg) for 2 hr at 37°C. The assay was terminated by the addition of ice cold Tris buffer followed by centrifugation at 1800 g for 15 min at 4°C. Radioactivity in the pellet was determined. Data was plotted as percentage inhibition (over maximum binding, obtained in the absence of antiserum) as a function of antiserum dilution.

#### Isotype analysis of elicited anti-hCG antibodies

Diluted sera of animals immunized with βhCG-TT and βhCG-TT + *MIP* were added to wells previously adsorbed with 100 ng hCG. Following a 16-hr incubation at 4°C, a 2-hr incubation was carried out with biotin-conjugated isotype specific antibodies (BD Biosciences). Incubation with streptavidin-HRP was carried out for 2 hr and enzyme activity visualized using a TMB substrate. The reaction was terminated with 2N H_2_SO_4_ and optical densities determined at 450 nm. Ratios of Relative Absorbance Units (products of O.D. and sera dilution at an appropriate sera dilution) were calculated for sera derived from animals immunized with βhCG-TT and βhCG-TT + *MIP*.

#### Reactivity of elicited anti-hCG antibodies towards tumor cells and effects on viability

For assessment of intracellular reactivity, cells were first permeabilized by 90-sec incubation in chilled methanol containing 0.001% v/v Triton X-100. Permeabilized and non-permeabilized SP2/O (murine myeloma; ATCC) and B16F10 (murine melanoma; ATCC) cells were incubated for 2 hr at 4°C with 1∶50 diluted sera obtained either from βhCG-TT, βhCG-TT + *MIP* or from non-immunized animals; negative controls included cells incubated with anti-TT or anti-*MIP* antisera. After incubation with a 1∶150 diluted anti-mouse FITC antibody (Jackson ImmunoResearch), cellular reactivity was assessed on a BD-LSR flow cytometer. Reactivity of antisera towards permeablized COLO205 (human colorectal cancer; ATCC) was assessed by indirect immunofluorescence; cells were incubated for 2 hr at room temperature with 1∶50 diluted sera obtained either from βhCG-TT, βhCG-TT + *MIP* or from non-immunized animals. After incubation with a 1∶150 diluted anti-mouse FITC antibody (Jackson ImmunoResearch), digital images were captured on a Nikon immunofluorescence microscope.

B16F10 (10^4^) and SP2/O (5×10^4^) cells were cultured for 50 hr with 1∶2 diluted antisera (previously heat-inactivated by incubation at 56°C for 30 min) derived from βhCG-TT or βhCG-TT + *MIP* immunized mice. 40 µl of 5 mg/ml MTT was dispensed and an incubation carried out for 5 hr. 100 µl of DMSO was then added and incubated for another hour, subsequent to which optical density was determined at 550 nm. For competition studies, 1 µg/ml of hCG was co-incubated with the cells along with the antisera.

### Effect of immunization on tumor growth

Female BALB/c mice (n = 16) were immunized at fortnightly intervals with βhCG-TT, *MIP*, or βhCG-TT + *MIP*, using the protocol described above; a fourth group of animals received no immunization. At Day 35 post-immunization, animals were subcutaneously implanted with 4 × 10^4^ SP2/O myeloma cells. Tumor volumes were assessed every alternate day using the formula 4/3πr^3^ where r = (l+w)/4, using the largest axis of the tumor (l, length; w, width).

### Statistical analysis

Statistical analysis was carried out using the Unpaired Student's t-test using SigmaPlot version 11.

## Results

### βhCG/hCG as a growth factor for tumor cells *in vivo*

**Figure 1:**
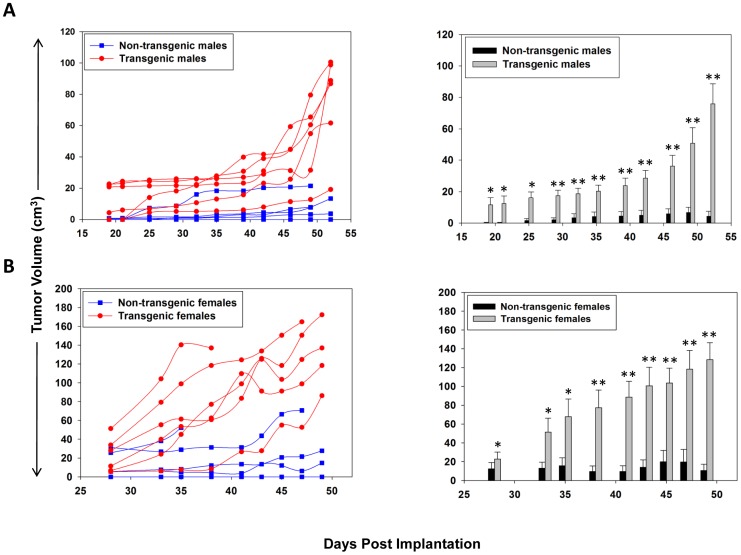
*In vivo* tumor-promoting potential of βhCG. SP2/O cells were implanted in BALB/c x FVB/J^βhCG/-^ F1 transgenic and non-transgenic male (A) and female (B) mice. Left panels depict tumor volumes in individual animals and right panels mean ± standard error, at different times post-cell implantation. *p≤0.05; **p≤0.01 versus non-transgenic littermates. n = 6.

The presence of the βhCG transgene was assessed in genomic DNA isolated from BALB/c x FVB/J^βhCG/-^ F1 mice. PCR analysis revealed a product of≅830 kb in about half the animals, as expected; βhCG was detected in the sera of animals harbouring the transgene (Figure S1). SP2/O murine myeloma cells (syngeneic for BALB/c) were subcutaneously implanted in transgenic and non-transgenic F1 mice of both sexes. In both male (Figure 1A) and female (Figure 1B) transgenic F1 mice, tumors grew at a significantly enhanced rate in comparison with non-transgenic littermates. These findings established that βhCG/hCG can act as a significant growth-promoting factor *in vivo*.

### Inflammatory effects of MIP on cells of the innate immune system

**Figure 2:**
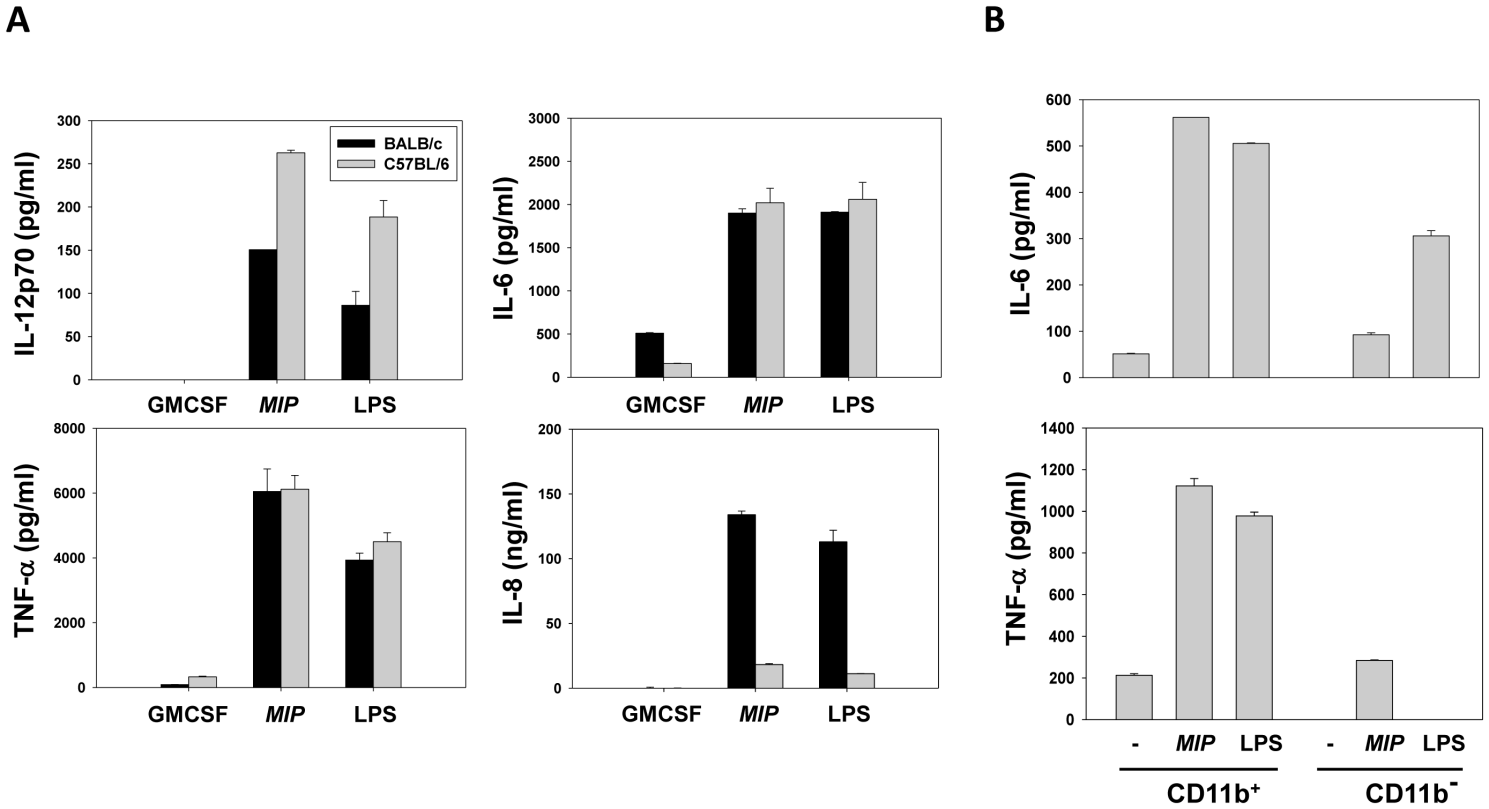
Inflammatory activity of MIP. (A) BMDC (derived from BALB/c and C57BL/6 mice) were treated with *MIP* (or LPS a control) and indicated cytokines were quantified. Bars represent arithmetic mean ± standard error. (B) IL-6 and TNF-α secretion upon incubation of MIP or LPS with CD11b^+^ and CD11b^−^ splenic cells derived from C57BL/6 mice.

The effect of *MIP* on the maturation of bone marrow-derived dendritic cells from BALB/c and C57BL/6 mice was analyzed. Culture supernatants isolated during this process demonstrated the elevated presence of inflammatory cytokines IL-12p70, IL-6, TNF-α and IL-8 in cells derived from both strains of mice (Figure 2A). *MIP* lysate also induced the secretion of IL-6 and TNF-α from CD11b^+^ splenic macrophages from C57BL/6 mice (Figure 2B).

### Comparative evaluation of immune responses upon immunization of βhCG-TT and βhCG-TT + *MIP* formulations

#### T cell recall responses

**Figure 3:**
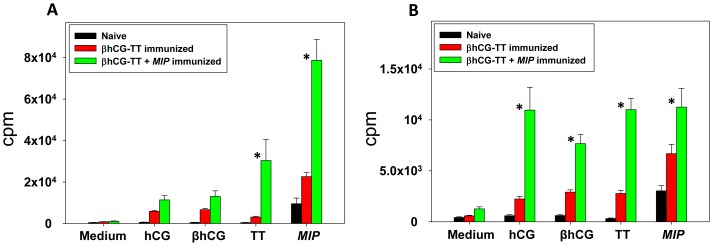
Proliferative T cell recall responses. *In vitro* proliferative responses to hCG, βhCG, TT or *MIP* of splenocytes derived from naive, βhCG-TT or βhCG-TT + *MIP* immunized (A) BALB/c and (B) C57BL/6 mice. Bars represent arithmetic mean ± standard error. *p≤0.05 versus vs animals immunized with βhCG-TT. n = 6.

**Figure 4:**
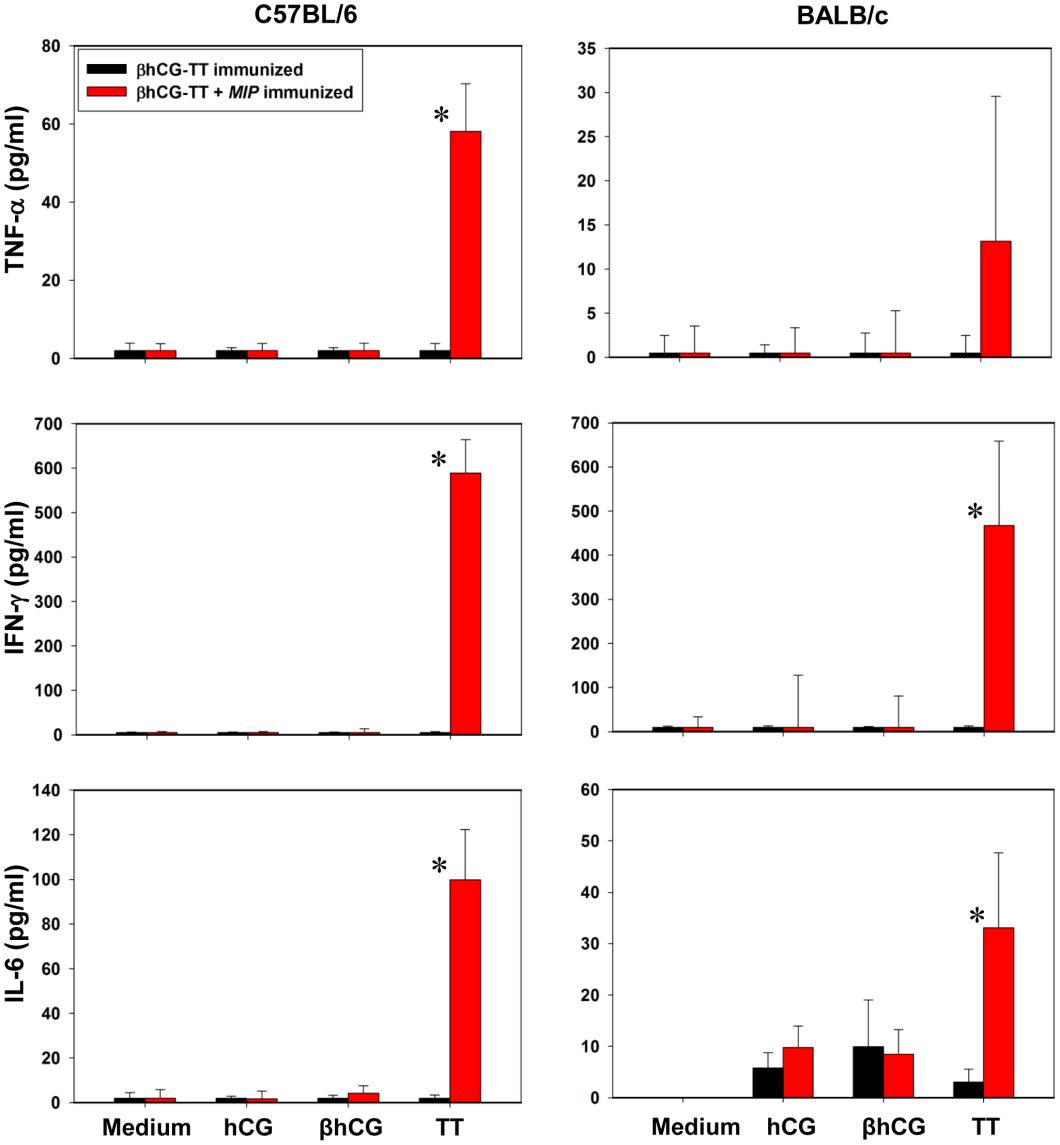
Cytokine recall responses. *In vitro* cytokine levels induced by hCG, βhCG or TT in splenocytes derived from βhCG-TT or βhCG-TT + *MIP* immunized BALB/c and C57BL/6 mice. Bars represent arithmetic mean ± standard error. *p≤0.05 versus animals immunized with βhCG-TT. n = 6.

*MIP* induced vigorous proliferative T cell recall responses in both BALB/c and C57BL/6 mice immunized with βhCG-TT + *MIP*. Interestingly, enhanced proliferative recall responses towards TT were observed in animals of both strains immunized with the *MIP*-supplemented formulation; hCG and βhCG recall responses were additionally heightened in C57BL/6 mice (Figure 3). In animals immunized with βhCG-TT + *MIP*, enhanced secretion of IFNγ and IL-6 in BALB/c mice, and of IFNγ, IL-6 and TNFα in C57BL/6 mice, was observed following *in vitro* incubation with TT (Figure 4). These results suggest that inclusion of *MIP* in the vaccine formulation leads to the superior *in vivo* priming of T cells recognizing vaccine components.

#### Anti-hCG antibody responses

**Figure 5:**
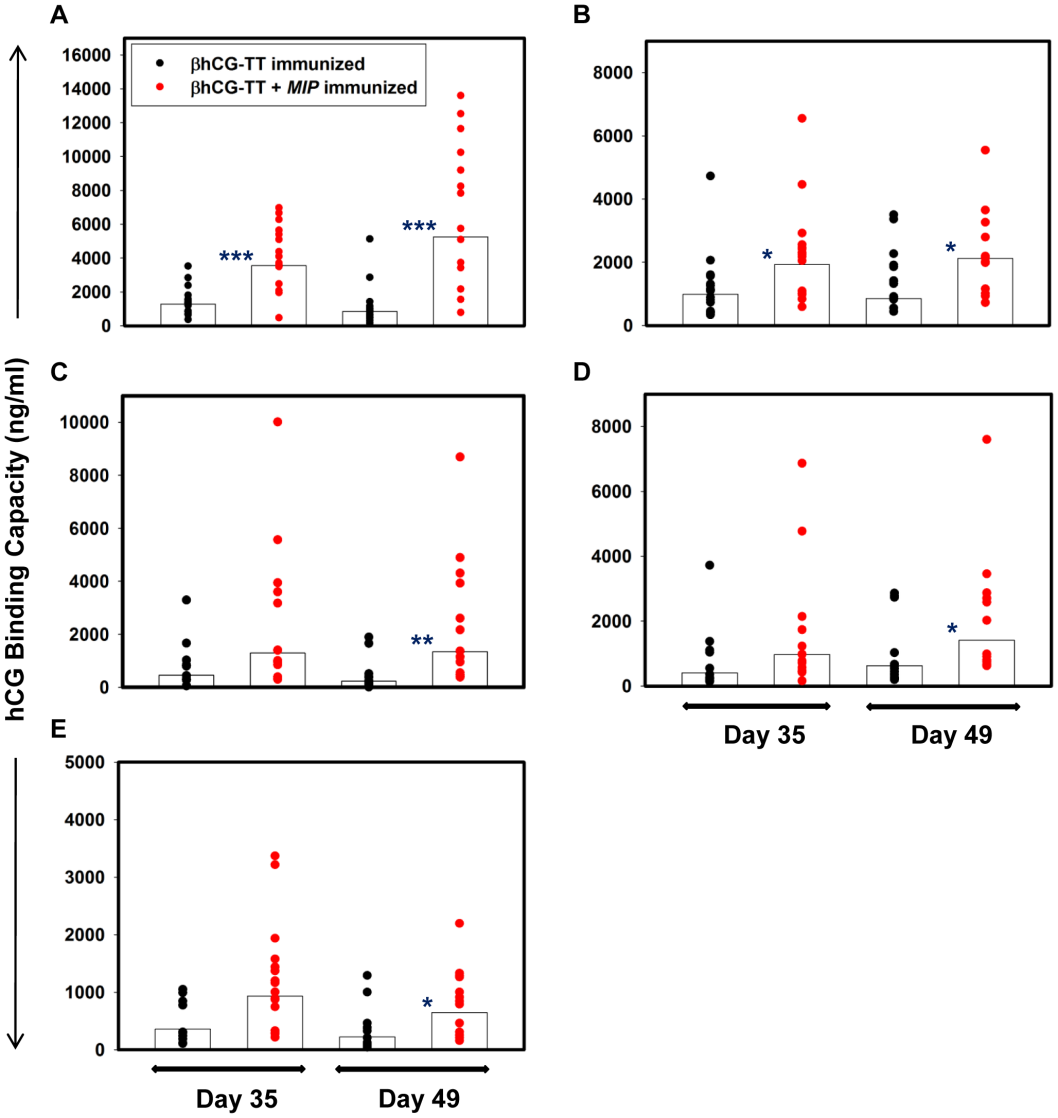
Immunogenecity studies. Immunogenicity of alum-adsorbed βhCG-TT and βhCG-TT + *MIP* in (A) BALB/c; (B) FVB/J; (C) C57BL/6; (D) CBA/Cat and (E) C3H/HeJ mice. Each point represents hCG binding capacity of an individual animal and bars denote geometric means. ***p≤0.001, **p≤0.01, *p≤0.05 versus βhCG-TT immunized animals. n = 16.

Incorporation of *MIP* into the βhCG-TT vaccine formulation resulted in significantly heightened anti-hCG antibody titres in all murine strains; increased antibody titres were observed at both Day 35 and Day 49 post-immunization (Figure 5). Elicited antibodies were found to possess high affinity (Ka≅10^10^ M^-^1) for hCG.

**Figure 6:**
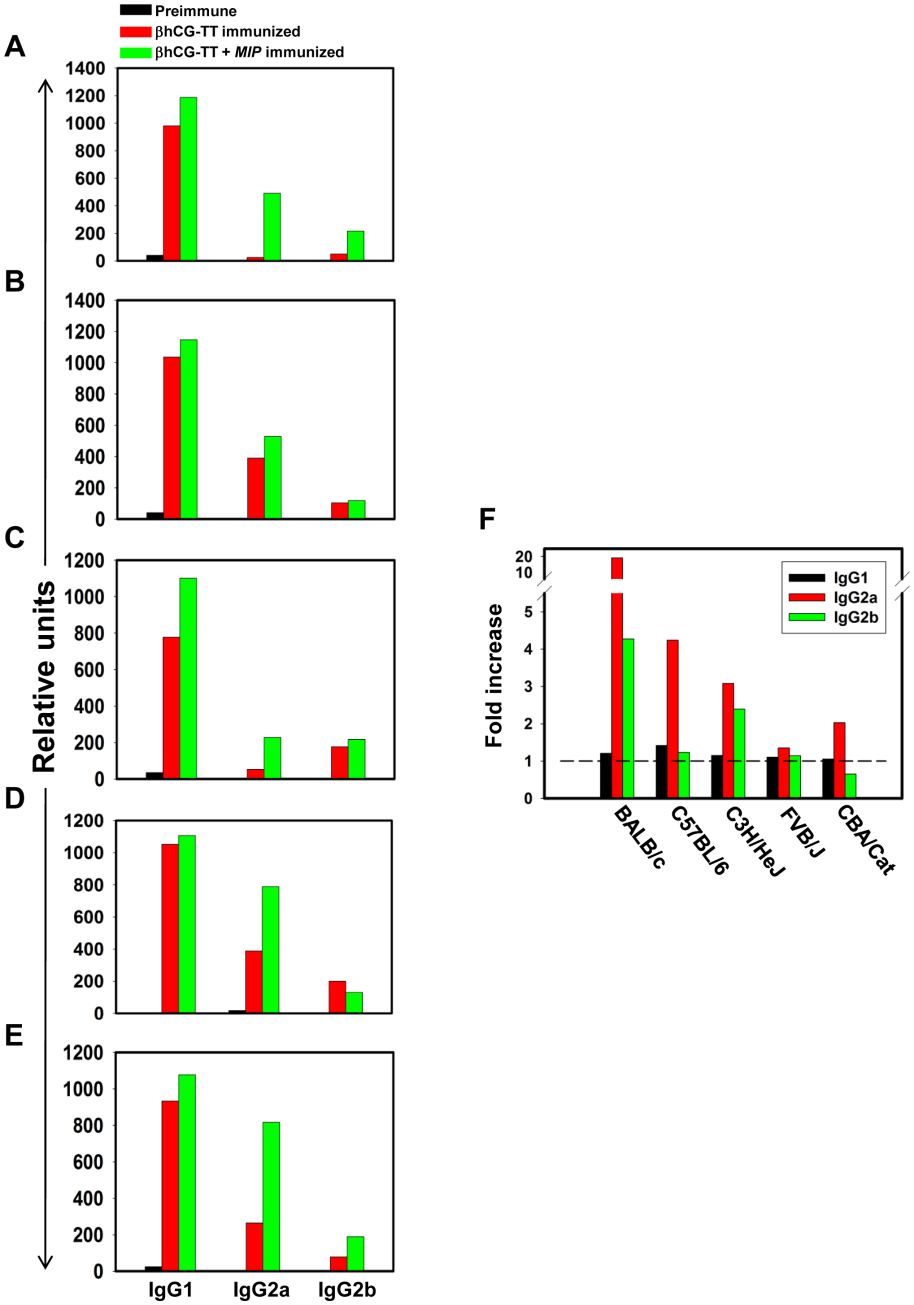
Anti-hCG antibody isotype analysis. Comparative anti-hCG immunoglobulin isotypic analysis of antibodies in five murine strains immunized with βhCG-TT and βhCG-TT + *MIP*. (A) BALB/c; (B) FVB/J; (C) C57BL/6; (D) CBA/Cat; (E) C3H/HeJ. Relative Units: Relative end-point titres. Pre-immune sera served as control. (F) Fold-increases in hCG specific IgG1, IgG2a and IgG2b antibody isotypes in different strains upon the inclusion of *MIP* in the vaccine formulation.

As an independent index of adjuvanticity, whether the incorporation of *MIP* influenced the isotypes of generated anti-hCG antibodies was assessed. Titres of individual isotypes were higher in animals receiving the *MIP*-supplemented formulation compared to those receiving βhCG-TT. Although the titres of anti-hCG antibodies of the IgG1 isotype were also higher in all murine strains upon the inclusion of *MIP*, the fold-increase was greater for IgG2a in four out of five strains, suggesting a modulation towards the Th1 response (Figure 6).

**Figure 7:**
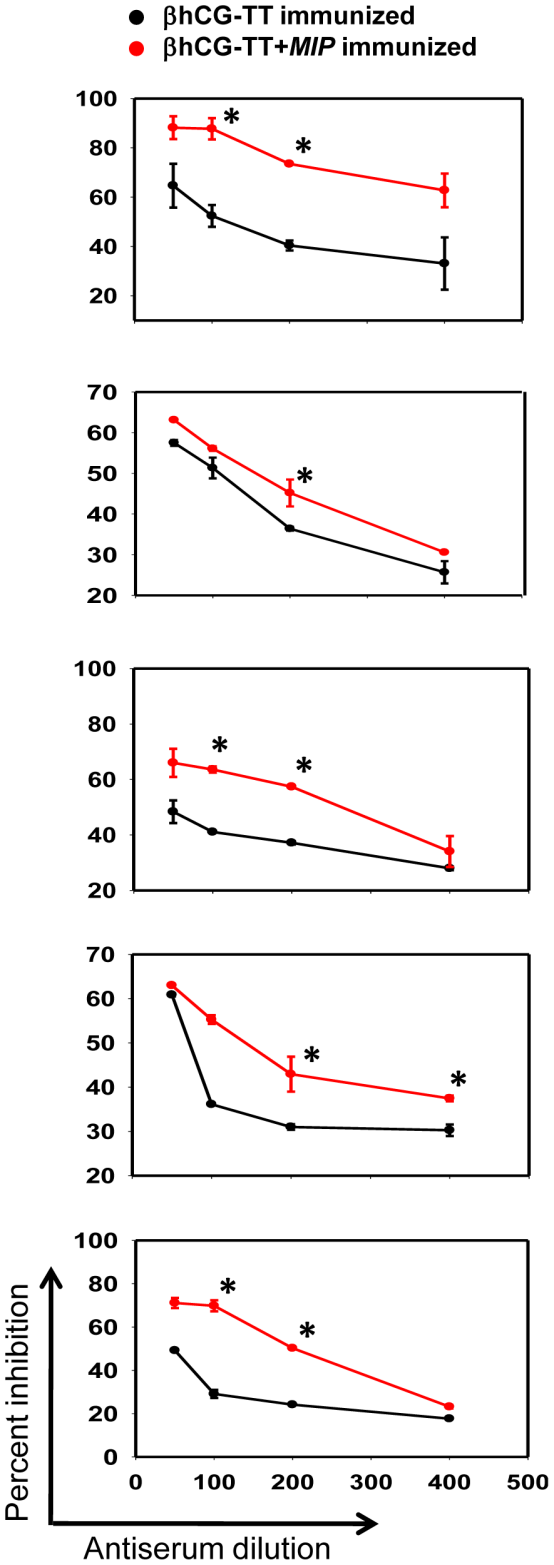
Bioneutralization potential of elicited antisera. hCG neutralization capacity of pooled antisera obtained from animals immunized with alum-adsorbed βhCG-TT or βhCG-TT + *MIP*, as determined by radio-receptor assay. (A) BALB/c; (B) FVB/J; (C) C57BL/6; (D) CBA/Cat and (E) C3H/HeJ. Data is presented as percentage inhibition (over maximal binding obtained in the absence of serum) as a function of antiserum dilution. *p≤0.05 versus corresponding value for βhCG-TT elicited antisera.

The ability of elicited antibodies to inhibit the interaction of hCG with its receptor was ascertained. The antibodies generated in animals immunized with the *MIP-*supplemented formulation were more potent in inhibiting hCG-receptor interaction; appreciable enhancements were seen in all five murine strains (Figure 7).

**Figure 8:**
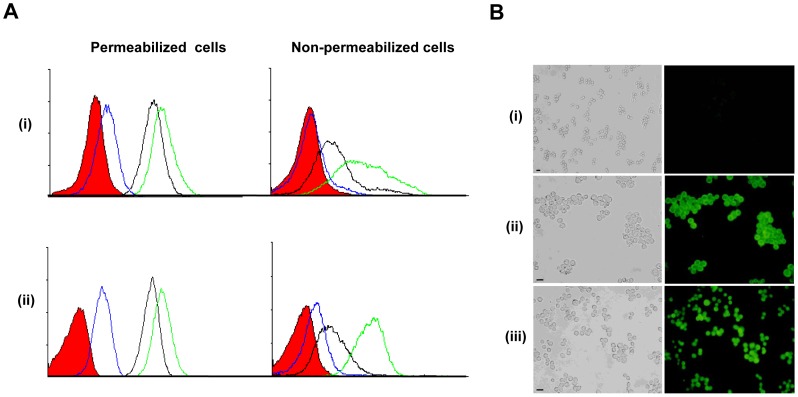
Reactivity of anti-hCG antibodies towards tumor cells. (A) Flow cytometric analysis on permeabilized and non-permeabilized (i) SP2/O and (ii) B16F10 cells using pooled antisera from βhCG-TT (black profiles) and βhCG-TT + *MIP* (green profiles) immunized mice; pre-immune sera (blue profiles) served as control. Filled red profiles: Secondary antibody controls. (B) Right panels: Immunofluorescence analysis on permeabilized COLO205 cells depicting reactivity of (i) pre-immune sera, (ii) antisera derived from βhCG-TT + *MIP* immunized mice and (iii) antisera derived from βhCG-TT immunized mice. Left panels: Corresponding phase contrast images. Bars = 30 µm.

Elicited anti-hCG antibodies were assessed for reactivity towards tumor cells. Flow cytometric analysis on SP2/O and B16F10 cells revealed enhanced recognition of cellular as well as surface moieties by antibodies in sera from βhCG-TT + *MIP* immunized mice in comparison with sera from βhCG-TT immunized mice (Figure 8A). Antibodies in antisera generated by the two formulations were reactive towards permeabilized COLO205 cells (Figure 8B), as well as towards other human cell lines, as assessed by indirect immunofluorescence.

**Figure 9:**
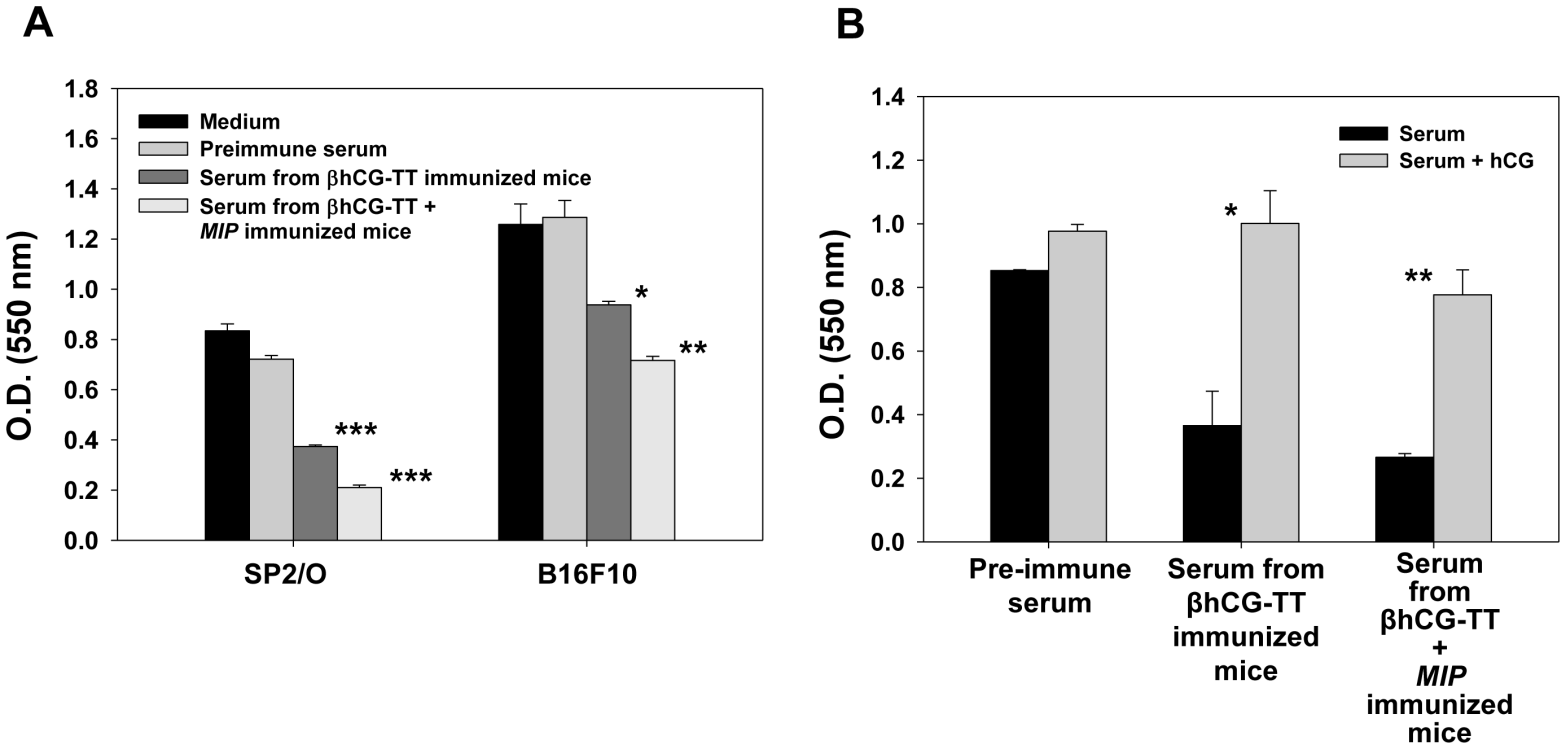
Effects of anti-sera on tumor cell viability. (A) The effects of pooled pre-immune serum and antiserum derived from mice immunized with βhCG-TT or βhCG-TT + *MIP* on the viability of SP2/O and B16F10 cells. Bars represent arithmetic mean ± standard error. *p≤0.05, **p≤0.01, ***p≤0.001 versus pre-immune serum. (B) Effect of co-incubation with hCG (1 µg/ml) on the loss of viability of SP2/O cells induced by antiserum from mice immunized with βhCG-TT or βhCG-TT + *MIP*. Bars represent arithmetic mean ± standard error. *p≤0.05, **p≤0.01versus serum alone.

Anti-hCG antibodies elicited upon immunization with the two formulations were assessed for cytotoxicity on SP2/O and B16F10 cells. Both cell lines demonstrated a loss of viability upon incubation with serum obtained from βhCG-TT and βhCG-TT + *MIP* immunized mice as opposed to incubation with serum obtained from pre-immune (naive) mice (Figure 9A). Pre-incubation with hCG negated detrimental effects on cell viability (Figure 9B).

### Effect of active immunization on the growth of syngeneic tumour cells

The effect of immunization with the βhCG-TT conjugate on growth of SP2/O myeloma-induced tumors in BALB/c mice was then assessed. Since *MIP* was observed to enhance anti-hCG titres when supplemented with the vaccine formulation, the bacterium was employed as an additional adjuvant in a second group of animals. A separate group of animals received only *MIP*, since the mycobacterium has been previously demonstrated to mediate independent anti-tumor effects in both mice and humans [14], [15], [19], [20].

**Figure 10:**
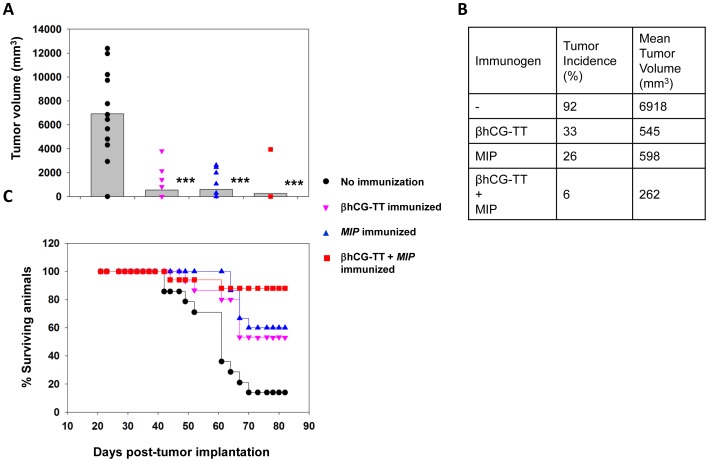
Effect of immunization on tumor volume, incidence and survival. (A, B) Tumor volume and incidence post-tumor implantation of SP2/O cells in BALB/c mice. Data on βhCG-TT, βhCG-TT + *MIP*, or *MIP-*immunized animals is depicted along with control (non-immunized) mice on Day 43 post-tumor cell implantation. In (A), each point represents an individual animal and bars denote geometric means. ***p≤0.001 versus the non-immunized group. (C) Survival curves for control animals and animals immunized with the different formulations, as indicated. n = 16.

Animals receiving either βhCG-TT or *MIP* immunization demonstrated significant decreases in tumour volume and incidence compared to control, non-immunized animals. Co-immunization of βhCG-TT and *MIP* provided additional benefit; mean tumour volumes as well as incidence were further reduced (Figure 10A, B). These results were substantiated by survival data. While only 14% of control animals survived at Day 82 post-tumor implantation, 53% of the animals immunized with βhCG-TT and 60% of the animals injected with *MIP* survived till this time. Significantly, 88% of the animals co-immunized with βhCG-TT and *MIP* survived (Figure 10C).

## Discussion

The unexpected association of hCG (or its subunits) with tumorigenesis [1] as well as its link to poor patient survival outcomes [2], [3] have sparked interest in strategies that target the molecule for the control of malignancy. Indeed, data published thus far from human studies indicates merit in the approach. A vaccine based on the C-terminal peptide (CTP) of βhCG has undergone Phase II trials in patients of metastatic colorectal cancer; anti-hCG antibody induction was shown to be associated with improved survival [12]. Constructs targeting βhCG to antigen presenting cells have been employed in patients with advanced epithelial cancers, in conjunction with TLR agonists [21].

Animal models have lent credence to the link between gonadotropin and tumorigenesis. In particular, mice transgenic for βhCG (FVB/J^βhCG/-^) develop obesity, pituitary prolactinomas and mammary gland adenocarcinomas, associated with excessive luteinization and infertility [16], [17]. Recent studies in our lab have described the preventive effects of anti-hCG immunization on the development of ovarian dysfunction and the induction of endogenous tumorigenesis in these animals [22]. In the current study, the growth-promoting potential of hCG/βhCG on implanted tumors was first established using a novel “self” *in vivo* model. In both male and female F1 transgenic mice, tumors grew at an enhanced rate compared to tumors in non-transgenic littermates upon the implantation of SP2/O cells, corroborating the role of hCG/βhCG in tumor progression. The fact that tumorigenesis is enhanced in both male and female βhCG transgenic mice tends to suggest a direct growth-promoting action of gonadotropin, though influence of gonad-derived steroids and further downstream events cannot be ruled out. Experiments underway in ovarectomized βhCG transgenic female mice will shed further light on the mechanistic aspects of the growth-promoting role of βhCG/CG on implanted tumors. To our knowledge, this is the first study which describes the promotion of implanted tumors of non-reproductive origin as a consequence of endogenous gonadotropin.

The targeting of CTP (instead of the entire βhCG subunit) has some theoretical merit as an anti-hCG vaccination strategy, in that generated antibodies are devoid of potentially problematic cross-reactivity to luteinizing hormone. Nevertheless, the current study employed the whole βhCG subunit, based on previous comparative analysis which revealed βhCG-based vaccines to be far superior to CTP-based vaccines in terms of immunogenicity, as well as in the bioneutralization capacity and association constants of the induced antibodies [10], [23]–[25]. Both strategies rely on chemical conjugation to carriers such as TT and DT to break tolerance toward hCG and enhance immunogenicity. The utility of targeting βhCG was demonstrated in pioneering Phase II clinical trials conducted by our lab [9]. Till date, these studies remain the only demonstration of the efficacy of a birth control vaccine in humans.

Clinical trials have suggested that both anti-fertility and anti-cancer applications of anti-hCG vaccination would benefit from enhanced immunogenicity. This study investigated the utility of *Mycobacterium indicus pranii* in this regard; the premise that its inclusion would not only act as a potent adjuvant for the generation of anti-hCG antibody responses but also mediate independent innate anti-tumor effects was based on published reports. The use of mycobateria in the treatment of cancer and other diseases has a long history. Work by other investigators has demonstrated that the immunogenicity of whole tumor cell vaccines can be enhanced by mycobacteria in animals and in humans [26]–[28]. BCG, as well as its cell wall skeleton (BCG-CWS) has been administered as adjuvant therapy in patients with colon, lung and bladder tumors [29]–[31]. An immunotherapeutic effect of *M. vaccae* administration was observed in patients with melanoma, advanced carcinoma of the prostate, lung cancer and renal cell carcinoma [32]–[35]. The anti-tumor efficacy of *MIP* has been associated with the induction of IFNγ [19] as well as a reduction in tumor-associated TReg cells [20] in mice.

Further rationale for the inclusion of *MIP* in the anti-hCG vaccine formulation came from its effects on antigen presenting cells in the current study. Enhanced secretion of inflammatory cytokines was observed in macrophages and BMDC cultures stimulated with *MIP*. Previous published work with other mycobacteria is in concordance with the observed immuno-stimulatory effects. *M. tuberculosis* up-regulated CD80, CD40 and CD54 levels and increased the secretion of TNFα and IL-12 [36], whereas BCG was seen to activate and cause maturation of DCs upon phagocytosis, while down regulating endocytic capacity and increasing TNFα production [37]. BCG-CWS has also been demonstrated to activate DCs by increasing the surface expression of co-stimulatory molecules and inducing the secretion of TNFα [38].

The reasons why proliferative responses to *MIP* appear to be higher in animals immunized with βhCG-TT, and indeed also somewhat elevated in naive animals as well, are as yet unclear. It can be hypothesized that the capability of *MIP* to elicit the secretion of cytokines from antigen presenting cells contributes to a favourable milieu in this regard *in vitro*, particularly for cells that have previously been activated. It is probable that this property contributes to MIP's adjuvant action.

Interestingly, the inclusion of *MIP* was found to enhance proliferative and cytokine T cell recall responses towards βhCG, hCG and TT in the current study. While the existence of T cell epitopes on TT is not unexpected, previous studies have also identified such epitopes on hCG in mice and rabbits [39]. That TT does not result in higher recall responses than hCG or βhCG in βhCG-TT immunized animals was unexpected and interesting. Given conventional wisdom, TT should act as “more non-self” than hCG or βhCG in mice, given its microbial origin. However, there has not as yet been a systematic study to hierarchically categorise anti-TT and anti-hCG T cell epitopes in mice. While the current study might be a first step in this regard, two factors should be borne in mind. Firstly, the nature of conjugation of βhCG to TT may affect T cell immunogenicity of one or both partners in ways which cannot be predicted *a priori*, and secondly, the data would not be translatable to humans, where hCG would constitute a “self” molecule, unlike in mice.

During recall assays in animals immunized with βhCG-TT + *MIP*, TT also induced significantly heightened levels of IFNγ and TNFα, cytokines with significant anti-tumor activity. It can be speculated that enhanced T cell reactivity towards the carrier molecule in animals receiving *MIP* is the result of superior activation of antigen-presenting cells. In one conceived scenario, B cells specific for βhCG would internalize the βhCG-TT conjugate and present TT-derived peptides to activated T cells. Higher T cell reactivity towards the carrier in animals receiving *MIP* can be postulated to subsequently dictate enhanced T cell “help” to the βhCG-specific B cells.

Experiments to assess immunogenicity, carried out in five different murine strains (including strains traditionally considered low responders in anti-hCG vaccination protocols) clearly demonstrated the beneficial effects of the inclusion of *MIP* as an additional adjuvant. Indeed, experiments aimed at dose optimization outlined above indicated that the presence of *MIP* could compensate for a lowered antigen dose. These observations are significant as they indicate efficacy across MHC haplotypes; vaccines intended for ultimate human use have to be necessarily designed for diverse populations. Enhancement in anti-hCG antibody titres upon the inclusion of *MIP* was invariably accompanied by enhancement in the bioneutralization capacity of the antibodies, indication that at least a sub-population of the antibodies targets the receptor-binding region of hCG. It is intriguing to speculate that the superior neutralization capacity of anti-hCG antibodies generated in response to βhCG-TT + *MIP* immunization could be attributable to factors other than titre. However, preliminary experiments designed to shed light on the issue (by assessing the hCG neutralization capacity of βhCG-TT + *MIP*-generated versus βhCG-TT-generated antisera normalized for titre) have indicated that increased titre probably plays a dominant role.

Incorporation of *MIP* enhanced IgG2a anti-hCG antibody titres to a relatively greater extent in comparison with IgG1 titres, reflecting a modulation towards a Th1 response; the shift occurred despite the concurrent presence of alum, an adjuvant known to promote a Th2 bias [13], [40]. Such effects have been previously reported to occur with other mycobacteria as well; for example, while ovalbumin induces Th2-type immune responses when administered intra-tracheally in immunized mice transgenic for a T cell receptor specific for ovalbumin, the concomitant presence of *M. tuberculosis* induces a Th1 skew [41]. Antibody isotypes induced by Th1 cytokines, namely IgG2a and IgG2b, are better at antibody-dependent cell-mediated cytotoxicity in mice [42], and macrophages were established to be the effector cells in tumoricidal effects mediated by monoclonal IgG2a antibodies in nude mice [43].

Anti-hCG antibodies in sera generated upon immunization were found to mediate complement-independent killing of tumor cells *in vitro*, although precise mechanisms remain unclear. hCG is known to inhibit apoptosis in the corpus luteum by upregulation of the anti-apoptotic protein Bcl-2 levels and downregulation of the pro-apoptotic protein Bax [44]. Its anti-apoptotic effects have also been demonstrated in ovarian cancer cells exposed to cisplatin [4]. For human tumors, anti-hCG antibodies could conceivable induce death by sequestering secreted CG, thereby depriving cells of its anti-apoptotic, growth-promoting effects. Alternatively, anti-hCG antibodies could bind CG (or other cross-reactive moieties) on the cell surface and transduce an apoptosis-inducing signal into the cell; these hypotheses require further validation.

In addition to providing substantial adjuvantic benefits, *MIP* also mediated significant independent anti-tumor effects. Synergistic benefits on tumor parameters and animal survival were observed when *MIP* and βhCG-TT were co-immunized. Strategies that activate both antigen specific (adaptive) and antigen non-specific (innate) arms of the immune system are being increasingly explored in immune-therapeutic regimens [45]. The results reported here suggest that use of additives such as *MIP* that mediate adjuvanticity to enhance specific anti-tumor antibody responses, as well as independently elicit innate anti-tumor effects, may be usefully employed for the treatment of gondotropin-secreting and/or sensitive cancers which frequently acquire resistance to conventional therapies.

## Supporting Information

Figure S1 **Evaluation of BALB/c x FVB/J^βhCG/-^ F1 mice.** (A) Detection of the βhCG transgene by PCR on genomic DNA. Individual mice are indicated by number. M: 100 bp marker. (B) Estimation of βhCG in the sera of the same animals by RIA.
(TIF)
