# Supplementary figures and images for: Synergistic Activation of Innate and Adaptive Immune Mechanisms in the Treatment of Gonadotropin-Sensitive Tumors
Source: PLoS One. 2013 Apr 8;8(4):e61288. doi: 10.1371/journal.pone.0061288 (PMC3620410; doi:10.1371/journal.pone.0061288)

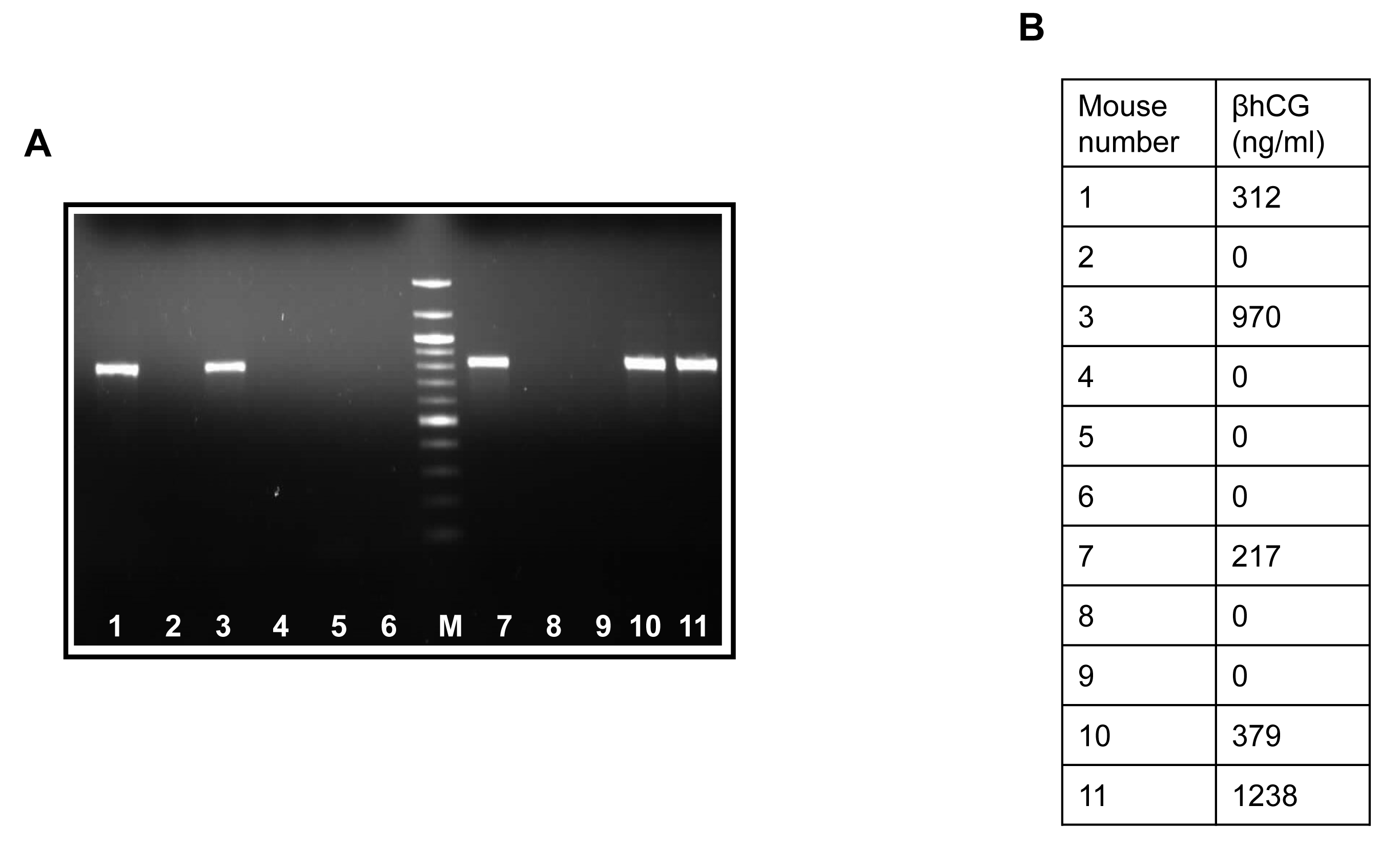

Supplement: Figure S1 — Evaluation of BALB/c x FVB/JβhCG/- F1 mice. (A) Detection of the βhCG transgene by PCR on genomic DNA. Individual mice are indicated by number. M: 100 bp marker. (B) Estimation of βhCG in the sera of the same animals by RIA. (TIF) [file pone.0061288.s001.tif]
